# Neurochemical characterisation of lamina II inhibitory interneurons that express GFP in the PrP-GFP mouse

**DOI:** 10.1186/1744-8069-9-56

**Published:** 2013-10-31

**Authors:** Noboru Iwagaki, Francesca Garzillo, Erika Polgár, John S Riddell, Andrew J Todd

**Affiliations:** 1 Institute of Neuroscience and Psychology, College of Medical, Veterinary and Life Sciences, University of Glasgow, Glasgow G12 8QQ, UK

## Abstract

**Background:**

Inhibitory interneurons in the superficial dorsal horn play important roles in modulating sensory transmission, and these roles are thought to be performed by distinct functional populations. We have identified 4 non-overlapping classes among the inhibitory interneurons in the rat, defined by the presence of galanin, neuropeptide Y, neuronal nitric oxide synthase (nNOS) and parvalbumin. The somatostatin receptor sst_2A_ is expressed by ~50% of the inhibitory interneurons in this region, and is particularly associated with nNOS- and galanin-expressing cells. The main aim of the present study was to test whether a genetically-defined population of inhibitory interneurons, those expressing green fluorescent protein (GFP) in the PrP-GFP mouse, belonged to one or more of the neurochemical classes identified in the rat.

**Results:**

The expression of sst_2A_ and its relation to other neurochemical markers in the mouse was similar to that in the rat, except that a significant number of cells co-expressed nNOS and galanin. The PrP-GFP cells were entirely contained within the set of inhibitory interneurons that possessed sst_2A_ receptors, and virtually all expressed nNOS and/or galanin. GFP was present in ~3-4% of neurons in the superficial dorsal horn, corresponding to ~16% of the inhibitory interneurons in this region. Consistent with their sst_2A_-immunoreactivity, all of the GFP cells were hyperpolarised by somatostatin, and this was prevented by administration of a selective sst_2_ receptor antagonist or a blocker of G-protein-coupled inwardly rectifying K^+^ channels.

**Conclusions:**

These findings support the view that neurochemistry provides a valuable way of classifying inhibitory interneurons in the superficial laminae. Together with previous evidence that the PrP-GFP cells form a relatively homogeneous population in terms of their physiological properties, they suggest that these neurons have specific roles in processing sensory information in the dorsal horn.

## Background

Lamina II of the spinal dorsal horn contains numerous densely-packed interneurons, which are involved in transmitting and modulating somatosensory information before it is conveyed to the brain (through projection neurons located in other laminae) and to deeper regions of the spinal cord [1]. Quantitative immunocytochemical studies in rat and mouse have suggested that approximately 30% of the interneurons in this lamina are GABAergic, with some using glycine as a co-transmitter [2-4], and these are thought to play an important role in suppressing pain and itch [5-8]. Numerous attempts have been made to identify distinct functional populations among the interneurons in this region, based on their morphological, physiological and neurochemical properties, or their developmental lineage [9-28]. The most widely accepted scheme is that developed by Grudt and Perl [11], which recognises four major classes of lamina II interneuron, defined mainly by their somatodendritic morphology: islet, vertical, central and radial cells. It has been shown that islet cells are inhibitory, while most vertical and radial cells are excitatory [14,15,19,20,29]. However, although these cell types have been recognised in other studies, a significant proportion of the lamina II neurons cannot be unequivocally assigned to any of these classes [11,12,14,15,23,30].

Neurochemistry provides a complementary way of classifying neurons, and we have identified four non-overlapping populations of dorsal horn inhibitory interneurons that express neuropeptide Y (NPY), galanin, neuronal nitric oxide synthase (nNOS) or parvalbumin, in the rat [17,31]. The somatostatin receptor sst_2A_ is restricted to GABAergic neurons in the superficial laminae, and is expressed by around half of these cells [4,13,14,32]. We have recently found that in the rat, most of the galanin and nNOS-expressing inhibitory interneurons showed sst_2A_-immunoreactivity, while this was present on only 16% of the NPY cells and virtually none of those with parvalbumin [13]. This suggests that the receptor is associated with specific functional classes of inhibitory interneuron. However, little is yet known about the relationship between these neurochemical populations in the mouse, which is now widely used in pain research, due to the availability of genetically modified lines.

Several recent studies have used mice in which fluorescent proteins are expressed in subsets of interneurons, in order to target these for whole cell patch-clamp recording [12,33-40]. One of these lines is the PrP-GFP mouse, in which enhanced green fluorescent protein (GFP) is expressed under control of the prion promoter [34,35,40,41]. GFP expression in the spinal cord of this mouse is largely restricted to lamina II, where it labels a set of inhibitory interneurons [35]. These cells form a physiologically homogeneous population, and show consistent features in terms of their synaptic inputs and outputs [34,35,40]. However, little is known about their expression of the neurochemical markers that have been identified in inhibitory interneurons, apart from the fact that they do not contain parvalbumin [34].

The initial aims of this study were to test whether the pattern of expression of sst_2A_ by cells that contained galanin, nNOS, NPY or parvalbumin was the same in the mouse as reported for the rat [13], and to determine whether the PrP-GFP cells corresponded to one or more of these neurochemical populations. Since we found that all of the PrP-GFP cells expressed sst_2A_ receptors, we went on to characterise their responses to somatostatin, specifically testing whether these were mediated by sst_2_ receptors. We also looked for involvement of G-protein-coupled inwardly rectifying K^+^ (GIRK) channels, which are thought to be activated by somatostatin in lamina II neurons [42].

## Methods

All animal experiments were approved by the Ethical Review Process Applications Panel of the University of Glasgow, and were performed in accordance with the European Community directive 86/609/EC and the UK Animals (Scientific Procedures) Act 1986.

### Neurochemistry of inhibitory interneurons

#### *Animals*

Nine PrP-GFP mice [41] of either sex (6–10 week old; University of Glasgow Biological Services) and 3 male NIHS mice (32-34 g; Harlan UK; closely related to the Swiss-Webster strain from which the PrP-GFP line was derived) were deeply anaesthetised with pentobarbitone (30 mg i.p.) and perfused through the left ventricle with fixative. For the PrP-GFP mice this contained 4% freshly de-polymerised formaldehyde, while for the NIHS mice it contained 4% formaldehyde/0.2% glutaraldehyde. The glutaraldehyde-containing fixative was used to maximise retention of GABA [43,44].

#### *General features of tissue processing, confocal microscopy and analysis*

Following perfusion fixation, the lumbar spinal cord was removed from all animals and the L4 segment was cut into transverse sections 60 μm thick with a vibrating microtome. All sections were immersed in 50% ethanol for 30 mins, and those from glutaraldehyde-fixed animals were treated with 1% sodium borohydride for 30 mins (to reduce free aldehyde groups), followed by extensive rinsing. Sections were then processed for multiple-labelling immunofluorescent detection, as described below. Details of the sources and concentrations of primary antibodies are given in Table 1. All secondary antibodies were raised in donkey and were species-specific. Secondary antibodies were conjugated to Rhodamine Red, DyLight 649 (1:100, 1:500, respectively; both from Jackson Immunoresearch, West Grove, PA, USA), or Alexa 488 (1:500; Life Technologies, Paisley, UK). In some cases, biotinylated secondary antibodies (1:500; Jackson Immunoresearch) were used, and these were revealed with avidin conjugated to Pacific Blue (1:1,000; Life Technologies). Primary antibody incubations were for 3 days and those in secondary antibodies were overnight (both at 4°C). Antibodies were diluted in PBS that contained 0.3% Triton-X100, except for reactions involving anti-sst_2A_, in which 5% normal donkey serum was included in both primary and secondary antibody solutions. Sections from the PrP-GFP mice that were used to estimate the percentage of neurons with GFP were incubated in propidium iodide as a nuclear counterstain following the immunoreaction [32]. All sections were mounted in anti-fade medium and stored at -20°C.

**Table 1:** Antibodies used in this study

**Antibody**	**Species**	**Dilution**	**Source**
GABA	rabbit	1:5,000	DV Pow
sst2A	guinea pig	1:2,000	Gramsch laboratories
GFP	mouse	1:100	Synaptic Systems
nNOS	sheep	1:2,000	PC Emson
Galanin	rabbit	1:1,000	Bachem
NPY	rabbit	1:1,000	Bachem
Parvalbumin	goat	1:500	Swant
Parvalbumin	rabbit	1:500	M Watanabe
NeuN	mouse	1:500	Millipore

To avoid observer bias, sections were selected for confocal scanning and analysis before immunofluorescence was examined. They were scanned with a Zeiss LSM710 confocal microscope (with Argon multi-line, 405 nm diode, 561 nm solid state and 633 nm HeNe lasers) through a 40× oil-immersion lens (numerical aperture 1.3) with the pin-hole set to 1 Airy unit. Overlapping fields to cover the whole of laminae I-III were scanned at 2 μm z-separation through the full thickness of the section, except for the analysis of GABA immunoreactivity and for the counts of GFP^+^ neurons (see below).

All quantitative analyses were carried out with Neurolucida for Confocal software (MBF Bioscience; Williston, VT, USA). The outline of the grey matter and the border between laminae II and III were drawn for the transverse sections, and the locations of immunoreactive cells were plotted onto these outlines. The position of the lamina II/III border was determined either from dark field scans, or from the ventral border of the plexus of sst_2A_-immunoreactive dendrites [4,32]. In some cases the lamina I/II border, identified in dark field scans, was also included. A stereological method was used to determine the proportion of superficial dorsal horn neurons that were GFP-positive (see below), but not for any of the analyses of cell counts in the z-stacks that were obtained from the full thickness of the sections. However, the sampling bias towards larger neurons in these analyses is likely to have been very small, as the section thickness (60 μm) was considerably larger than the cell bodies of the neurons that were being sampled.

#### *GABA, sst_2A_ and nNOS*

We have recently demonstrated that sst_2A_ is restricted to GABA-immunoreactive neurons in laminae I-II of C57Bl/6 mice [4]. To confirm that this arrangement applied to NIHS mice (which are closely related to the PrP-GFP line) and to assess the extent of sst_2A_ expression among nNOS-containing inhibitory interneurons, we reacted sections from the 3 NIHS mice with rabbit anti-GABA, guinea pig anti-sst_2A_ and sheep anti-nNOS, and revealed these with fluorescent secondary antibodies.

The relationship between sst_2A_ and GABA was examined in 4 dorsal horns from each of 3 mice. Since penetration of GABA immunostaining is extremely limited in sections that have been reacted by this method [4,45,46], only the upper surface of the section was scanned, at 0.5 μm z-separation. Overlapping z-stacks to cover the whole of laminae I-III were obtained. All sst_2A_^+^ cells in laminae I-III that were present at the section surface (i.e. on the first optical section in the z-series that contained tissue) were initially identified and plotted onto a dorsal horn outline, and these cells were then examined for the presence of GABA-immunoreactivity.

Since nNOS is expressed by both inhibitory and excitatory interneurons [45], we used these sections to determine the proportion of inhibitory (GABA-immunoreactive) nNOS^+^ neurons that possessed sst_2A_ receptors. For this part of the study, 8 dorsal horns from each mouse were analysed, in order to provide a sufficient sample size. All nNOS^+^/GABA^+^ cells with nuclei at the section surface were identified and their locations were plotted. The presence or absence of sst_2A_ was then assessed for each of the selected cells.

#### *Neurochemical markers in PrP-GFP mice and their expression by GFP^+^ cells*

Sections from the PrP-GFP mice were used to analyse: (i) the expression of sst_2A_ by neurons immunoreactive for galanin, NPY or parvalbumin, (ii) expression of sst_2A_ by GFP^+^ neurons, (iii) the presence of nNOS, galanin, NPY or parvalbumin in GFP^+^ neurons, and (iv) the relationship between galanin and nNOS in sst_2A_^+^ (inhibitory) interneurons. The sections were reacted with mouse anti-GFP and guinea pig anti-sst_2A_, together with either (i) rabbit anti-galanin and sheep anti-nNOS, (ii) rabbit anti-NPY (in some cases combined with goat anti-parvalbumin), or (iii) rabbit anti-parvalbumin. These were revealed with appropriate fluorescent secondary antibodies. From each of four mice, we scanned four dorsal horns that had been reacted with the galanin/nNOS antibody combination, and four dorsal horns reacted for NPY and parvalbumin.

To investigate expression of sst_2A_ among the different neurochemical populations, all cells in laminae I-III that were immunoreactive for galanin, NPY or parvalbumin were plotted, and then the sst_2A_ channel was viewed and the presence or absence of the receptor was noted for each of these neurons. Expression of sst_2A_ by GFP^+^ cells was analysed in 5 mice (4–8 dorsal horns from each mouse). The GFP channel was initially viewed and the locations of all GFP^+^ cells in laminae I-II were plotted. The sst_2A_ channel was then examined and the presence or absence of sst_2A_ staining was noted for each of the GFP^+^ cells.

To determine whether GFP^+^ cells contained nNOS, galanin, NPY or parvalbumin, we looked for the presence of these types of immunoreactivity in all of the GFP^+^ cells identified in 4 dorsal horns from each of 4 mice for each neurochemical type.

During this part of the study, we observed that unlike the situation in the rat [31], there was significant coexistence of galanin and nNOS in sst_2A_-expressing neurons in laminae I-II. However, cells that were strongly galanin-immunoreactive were generally weakly stained for nNOS, and *vice versa*. We therefore analysed this coexistence quantitatively in two of the dorsal horns from each of four mice. To do this, the sst_2A_ and galanin channels were viewed, and all of the sst_2A_-expressing cells were identified. All of the cells that were galanin-immunoreactive were then assigned a score for the intensity of galanin-immunoreactivity, ranging from 4 (very strong) to 1 (weak) or 0 (negative). The sections were then re-examined, in this case with the channels for sst_2A_ and nNOS, and sst_2A_^+^ cells that were nNOS immunoreactive were assigned a score of 4 (very strong) to 1 (weak) or 0 (negative) to indicate the intensity of the nNOS immunoreactivity. The resulting Neurolucida files were then combined to reveal the strength of galanin and nNOS immunostaining for each of the cells that contained either or both of these substances.

Many of the cells with galanin and/or nNOS were found to be GFP^+^, and the intensity of GFP expression differed among these neurochemical groups. We therefore recorded the strength of GFP immunoreactivity in all GFP^+^ cells identified in these sections and related this to the pattern of galanin and nNOS expression. As before, we assigned a score of 4 (very strong) to 1 (weak) for GFP. This was done by an observer who was blind to the strength of galanin or nNOS immunoreactivity in these cells. As reported previously [35], occasional GFP^+^ cells were found below lamina II, but these were not included in any of the analyses.

#### *Proportion of superficial dorsal horn neurons with GFP in PrP-GFP mice*

In order to estimate the proportion of superficial dorsal horn neurons that were GFP^+^, we examined sections from 3 PrP-GFP mice that had been reacted with mouse anti-GFP, and then with NeuN antibody (revealed with Alexa 488 and DyLight 649, respectively), followed by propidium iodide. Four dorsal horns from each of these animals were scanned to produce z-series of 24 optical sections at 1 μm z-separation. These were analysed by using a modification of the optical disector method [32] as described in detail by Polgár et al. [47].

#### *Characterisation of antibodies*

The GABA antibody was raised against GABA conjugated to porcine thyroglobulin with glutaraldehyde, and shows negligible cross-reactivity against other amino acids (glutamate, aspartate, glycine or taurine) [48]. The sst_2A_ antibody was raised against the C terminal 15 amino acids of the peptide sequence of the rat and mouse sst_2A_ receptor, coupled to keyhole limpet haemocyanin. Immunostaining was blocked by incubation with the peptide antigen (manufacturer’s specification). The GFP antibody was raised against recombinant GFP, and the distribution of immunostaining matched that of cells that expressed GFP. The nNOS antibody labels a band of 155 kDa in Western blots of rat hypothalamus, and staining is abolished by pre-incubation with nNOS [49]. We have reported that dorsal horn immunostaining with the galanin and NPY antibodies can be abolished by pre-treatment with the corresponding peptides [50,51], and staining of neurons with the galanin antibody is absent from the brains of galanin knock-out mice [52]. The rabbit and goat parvalbumin antibodies were raised against mouse and rat parvalbumin, respectively, and recognise a protein band of the appropriate size on Western blots. The NeuN antibody was raised against cell nuclei extracted from mouse brain and found to react with a protein specific for neurons [53]. We have shown that NeuN labels all neurons but does not label glial cells in the rat spinal dorsal horn [32].

### Responses of PrP-GFP cells to somatostatin

Young adult (4–6 week old) PrP-GFP mice (University of Glasgow Biological Services) were anaesthetised with continuous inhalation of 1-3% isoflurane. After laminectomy, the mid-thoracic to sacral spinal cord was removed and transferred to ice-cold dissecting solution containing: (mM) 3.0 KCl, 1.2 NaH_2_PO_4_, 2.4 CaCl_2_, 1.3 MgCl_2_, 26.0 NaHCO_3_, 15.0 glucose, 251.6 sucrose, oxygenated with 95% O_2_ and 5% CO_2_. The mouse was killed by anaesthetic overdose and decapitation. The dura and pia mater were removed, together with all dorsal and ventral roots. The isolated spinal cord was placed on an agar block positioned on a vibrating blade microtome (Microm HM 650 V, Fisher Scientific, Loughborough, UK). Parasagittal slices (200–300 μm) were cut, and these were subsequently kept for at least 30 min in recording solution containing: (mM) 125.8 NaCl, 3.0 KCl, 1.2 NaH_2_PO_4_, 2.4 CaCl_2_, 1.3 MgCl_2_, 26.0 NaHCO_3_, 15.0 glucose, oxygenated with 95% O_2_ and 5% CO_2_.

Slices were transferred to a recording chamber perfused with the oxygenated recording solution (flow rate ~2 ml/min) at room temperature. Whole-cell patch-clamp recordings were made from GFP-positive neurons. The targeted cells were visualised under infrared differential interference contrast and fluorescence microscopy on an Olympus BX51WI microscope. Patch electrodes were pulled with a horizontal puller (Sutter Instrument, Novato, CA, USA) from thin wall glass capillaries (World Precision Instruments, Sarasota, FL, USA). The electrode was filled with internal solution containing: (mM) 130 potassium gluconate, 10 KCl, 2 MgCl_2_, 10 HEPES, 0.5 EGTA, 2 ATP-Na_2_, 0.5 GTP-Na, pH adjusted to 7.3 with 1 M KOH. Neurobiotin (0.2%, Life Technologies) was also included in the internal solution for morphological analysis of recorded cells (to be reported subsequently). Typical electrical resistance of solution-filled electrodes was 4–6 MΩ. Patch-clamp signals were amplified and filtered (4 kHz low-pass Bessel filter) with a MultiClamp 700B amplifier (Molecular Devices, Sunnyvale, CA, USA) and acquired at 10 kHz using a Digidata 1440 A A/D board and pClamp 10 software (Molecular Devices). Acquired data were analysed using pClamp 10 software, and data are reported as mean ± SEM.

Drugs were bath-applied via 3-way stopcocks without any change in perfusion rate or temperature. They were purchased as follows: somatostatin (Merck Chemicals, Nottingham, UK), CYN 154806 (Tocris Bioscience, Bristol, UK), and tertiapin-Q (Abcam, Cambridge, UK).

Although the mice used for electrophysiology were slightly younger than the PrP-GFP mice in the anatomical part of the study, this should have no effect on the responses to somatostatin, as we have found that the pattern of sst_2A_ expression is already established within the first postnatal week in mice (AJT and EP, unpublished data).

### Statistical tests

Spearman’s rank correlation test was used to investigate the relationship between galanin and nNOS staining intensity among sst_2A_-expressing cells in the PrP-GFP mice, and Student’s t-test for electrophysiological data. P values of < 0.05 were taken as significant.

## Results

### Neurochemistry of interneuron populations

The distribution of immunostaining for nNOS, galanin, NPY, parvalbumin, sst_2A_ and GABA in the mouse spinal cord was very similar to that seen in the rat, although as reported by Hughes et al. [36], parvalbumin-immunoreactive lamina III cells were more numerous than in the rat.

**Figure 1:**
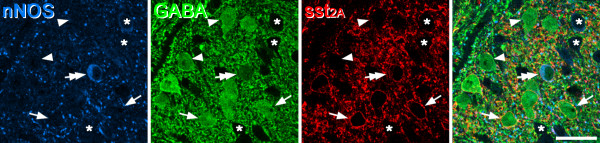
**nNOS, GABA and sst**_**2A **_**in lamina II in the mouse.** A single confocal scan through a section that had been reacted to reveal nNOS, GABA and sst_2A_. A nNOS^+^/GABA^+^ neuron (double arrow) is sst_2A_^+^. Several other GABA^+^ cells that lack nNOS are present in this field. Two of these that are sst_2A_^+^ are marked with arrows, while two that are sst_2A_^-^ are indicated with arrowheads. Three nearby GABA-negative cells are labelled with asterisks. Scale bar: 20 μm.

Between 104 and 132 (mean 118) sst_2A_^+^ cells were identified in laminae I-II in the sections from the 3 glutaraldehyde-fixed mice, and all but one of these cells (99.7%) were GABA-immunoreactive (Figure 1). Fourteen sst_2A_^+^ cells (mean 4.7/mouse) were present in lamina III in the sections analysed, and 10 of these (mean 67.5%) were GABA-immunoreactive.

**Figure 2:**
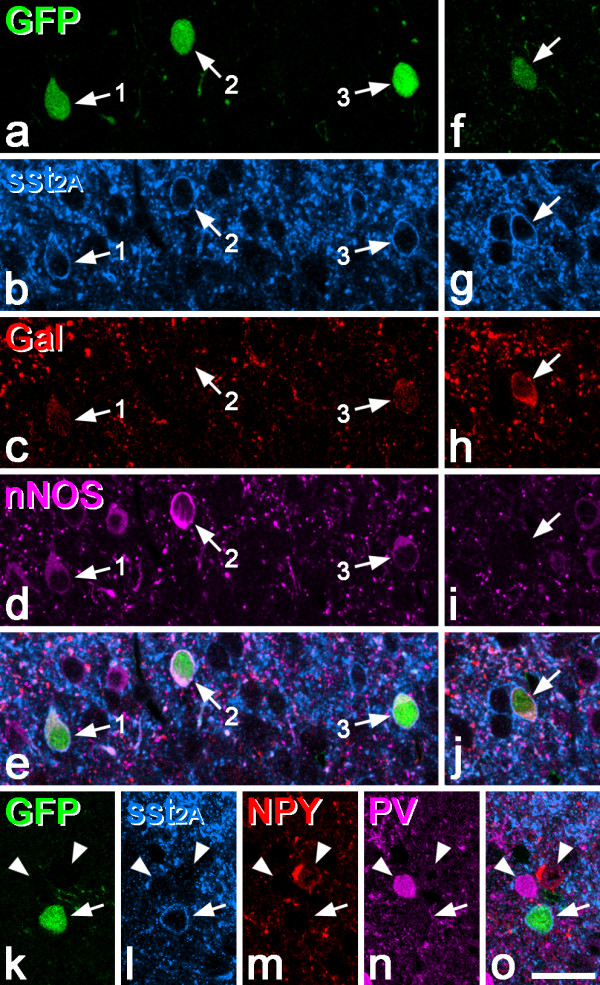
**GFP and sst**_**2A **_**expression among different neurochemical interneuron populations in the PrP-GFP mouse. a-e**, a field from lamina II contains three GFP-labelled neurons, all of which are sst_2A_^+^. The cells numbered 1 and 3 show weak immunoreactivity for both nNOS and galanin, while the cell numbered 2 is strongly nNOS^+^ and galanin^-^. **f-j**, a nearby region from the same section includes a cell with weak GFP (arrow) that is sst_2A_^+^ and shows strong galanin immunoreactivity, but lacks nNOS. **k-o**, This field from lamina II includes a GFP cell (arrow) that is sst_2A_^+^ but does not contain NPY or parvalbumin (PV). Two nearby cells are indicated with arrowheads. The one on the left is PV^+^/NPY^-^, while the one on the right is PV^-^/NPY^+^. Both of these cells lack sst_2A_. Scale bar (in o): 20 μm.

The relationship of sst_2A_ to galanin, nNOS/GABA, NPY and parvalbumin in the mouse was generally very similar to what we have reported previously in the rat [13] (Table 2, Figure 1, Figure 2). In laminae I-II, sst_2A_ was expressed by virtually all galanin^+^ and most (95%) nNOS^+^/GABA^+^ cells, but only by 24% of NPY^+^ cells and no parvalbumin^+^ cells. In lamina III, the receptor was expressed by 42% of the nNOS^+^/GABA^+^ cells and by a few galanin cells.

**Table 2:** **Expression of sst**_**2A **_**by different neurochemical types of interneuron in the mouse dorsal horn**

	**Laminae I + II**		**Lamina III**	
	**Number counted**	**% sst**_**2A**_	**Number counted**	**% sst**_**2A**_
nNOS/GABA	62.7	94.9	8.3	42.1
	(56–67)	(91–98.2)	(6–12)	(33.3-50)
Galanin	202.3	99.8	3	10.4
	(194–219)	(99.5-100)	(1–6)	(0–25)
NPY	95.6	24.2	28.5	4.1
	(73–109)	(17.4-31.5)	(21–34)	(0–6.9)
Parvalbumin	18.8	0	162.8	0
	(15–25)		(144–183)	

In each case the mean values for 3 NIHS mice (nNOS/GABA) or 4 PrP-GFP mice (galanin, NPY, parvalbumin) are shown, with the range in brackets.

Since GABA immunostaining could only be assessed at the top surface of the sections, there could be a sampling bias towards larger cells in this part of the analysis. However, since GABA was detected in 99.7% of sst_2A_ cells in laminae I-II, while 95% of GABA^+^/nNOS^+^ cells in this region were sst_2A_-immunoreactive, sampling bias is unlikely to have affected our interpretation.

**Figure 3:**
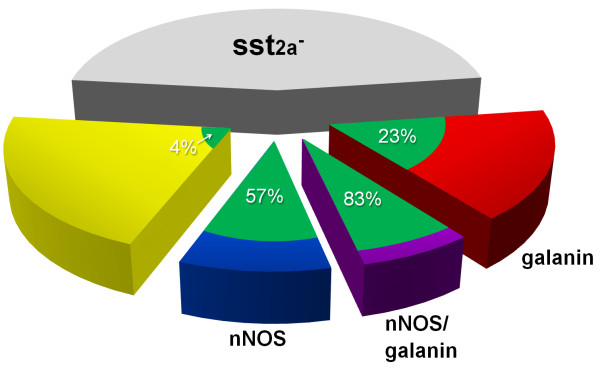
**Expression of galanin and nNOS among sst**_**2A**_^**+ **^**neurons in the superficial dorsal horn, and their relationship with GFP in the PrP-GFP mouse.** The pie chart shows the sizes of different neurochemical populations among the inhibitory interneurons in laminae I-II. We have estimated that 54% of the inhibitory interneurons in this region are sst_2A_-immunoreactive in a different mouse strain (C57Bl/6) [4], and the present results indicate that the proportion of these cells that contain only nNOS, only galanin or both nNOS and galanin are 17%, 31% and 13%, respectively. The proportions of each of these populations that are accounted for by GFP^+^ neurons in the PrP-GFP mouse are shown in green, with the corresponding percentages indicated.

**Figure 4:**
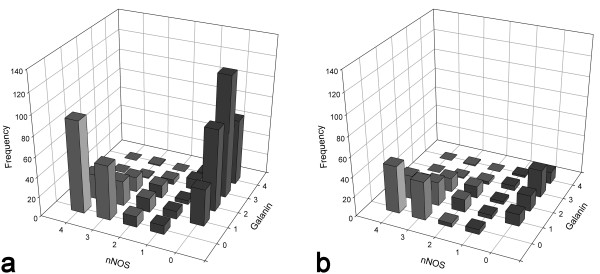
**Strength of immunostaining for nNOS and galanin among sst**_**2A**_^**+ **^**and GFP**^**+ **^**neurons in laminae I-II of the PrP-GFP mouse. a**, The frequency of cells with different intensities of labelling for nNOS and galanin, graded from 4 (strong) to 1 (very weak), or 0 (negative). Data were pooled from 4 mice (two dorsal horns from each mouse). Although many cells were only immunoreactive for nNOS or galanin, some showed both types of immunoreactivity. **b**, The equivalent frequency histogram for all of the cells shown in **a** that were also GFP-labelled.

As stated above, there was some coexistence of galanin and nNOS expression among sst_2A_^+^ cells in laminae I-II in the mouse (Figure 2a-e), unlike the situation in the rat [31]. Among the sst_2A_ cells, 17% (range 14-18%, n = 4 mice) were nNOS^+^/galanin^-^, 31% (24-36%) were nNOS^-^/galanin^+^, 13% (10-17%) were nNOS^+^/galanin^+^, while 39% (36-44%) contained neither galanin nor nNOS (Figure 3). The strength of immunostaining for nNOS and galanin among the sst_2A_^+^ cells that expressed one or both of these markers is shown in Figure 4a. There was a clear inverse relationship between the staining intensity for each marker (R_S_ = -0.76, P < 0.001, Spearman’s rank order correlation test).

### GFP neurons in the PrP-GFP mouse

In the PrP-GFP mouse, many GFP^+^ cells were present in the dorsal horn and these were largely restricted to lamina II, with occasional cells in laminae I and III, as reported previously [34,35,40]. Altogether, 1133 GFP^+^ neurons in the superficial dorsal horn (laminae I and II) were examined in sections from 5 mice (133–302 neurons per mouse) and all but one of these cells (99.9%, range 99.7-100%) were sst_2A_^+^ (Figure 2).

We next examined the expression of GFP among the different neurochemical populations. In the sections reacted for GFP, sst_2A_, galanin and nNOS, 98% (range 97-99%, n = 4 mice) of the GFP cells were nNOS- and/or galanin-immunoreactive (Figures 2a-j). Specifically, 35% (23-41%) were nNOS^+^/galanin^-^, 28% (23-33%) were nNOS^-^/galanin^+^, and 35% (29–41) were nNOS^+^/galanin^+^. In contrast, none of the GFP^+^ cells that were seen in the sections reacted for NPY or parvalbumin contained either of these markers (Figure 2k-o), consistent with the finding by Hantman and Perl [34] that the GFP cells are different from those that contain parvalbumin.

The relationship between GFP, galanin, nNOS and sst_2A_ was analysed in detail in 2 dorsal horns each from 4 mice (selected from the sections used to investigate coexistence of galanin and nNOS). The GFP cells constituted 28.8% (25.5-31.2%) of all sst_2A_ neurons in laminae I and II. When considering the different sub-populations of sst_2A_-expressing neurons, GFP cells accounted for 23% (15.9-33.3%) of those that contained galanin but not nNOS, 57% (44.4-67.4%) of those that contained nNOS but not galanin, 83% (66.7-90.7%) of the cells that contained both nNOS and galanin, and 4% (1.9-6%) of those that contained neither (Figure 3, Figure 4b). GFP was expressed by 69.2% (62.1-73.5) of all nNOS^+^/sst_2A_^+^ cells and by 41% (27.4-57.3) of all galanin^+^/sst_2A_^+^ cells in laminae I-II.

**Figure 5:**
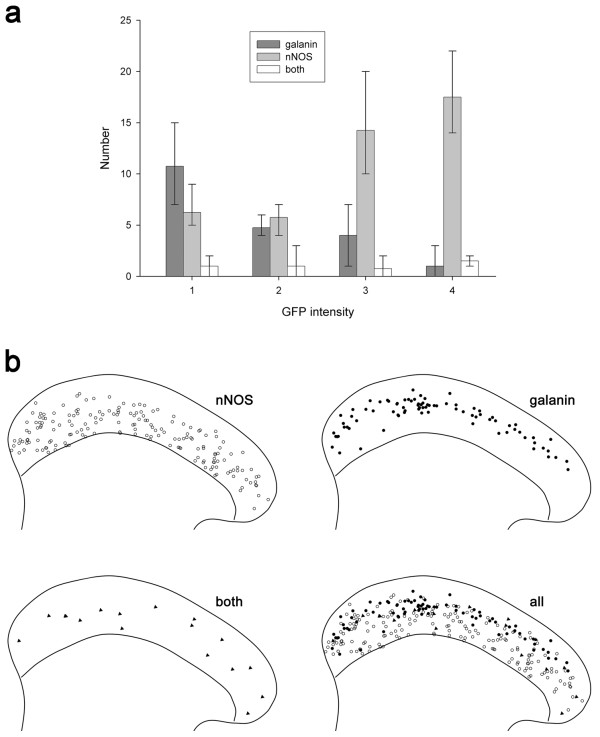
**GFP intensity among galanin and nNOS cells in the superficial dorsal horn of the PrP-GFP mouse and their laminar distribution.** The GFP^+^ cells that contained either galanin or nNOS were divided into 3 groups: those that had a higher intensity score for galanin (galanin), those with a higher score for nNOS (nNOS) and those for which the scores were equal (both). **a** The numbers of cells belonging to each group that were defined as having weak (1), medium (2), strong (3) or very strong (4) labelling for GFP. Each column shows the mean number of cells per mouse and the range across the 4 mice. Note that cells in the galanin group tended to have a low level of GFP, while those in the nNOS group often had high GFP levels. **b** The laminar location of cells in these 3 groups plotted onto an outline of the superficial dorsal horn. In each drawing the upper line represents the edge of the dorsal horn and the lower line the lamina II-III border. Cells belonging to the nNOS group are shown as open circles, those in the galanin group as filled circles and those defined as both as filled triangles. The lower right drawing shows all 3 groups combined.

The intensity of GFP labelling varied considerably among these cells, and this was related to their expression pattern for galanin and nNOS, and also to their location within lamina II (Figure 5). To simplify this part of the analysis, we divided the cells into 3 groups: those that were galanin^+^/nNOS^-^, or for which the galanin intensity score was higher than that for nNOS (galanin group), those that were galanin^-^/nNOS^+^ or for which the nNOS intensity score was higher than that for galanin (nNOS group) and the few cells for which the intensity scores for galanin and nNOS were equal ("both" group). As can be seen from Figure 5, cells belonging to the galanin group tended to have a lower intensity of GFP and to be located dorsally, while those in the nNOS group generally showed stronger GFP labelling and were located more ventrally within lamina II.

In the sections reacted with anti-GFP, NeuN and propidium iodide, between 676 and 756 (mean 710) neurons in laminae I and II were included in the disector sample from the 3 mice, and 3.4% (3.3-3.7%) of these were GFP^+^. When laminae I and II were analysed separately, between 71–88 neurons in lamina I were included and none of these were GFP^+^, while for lamina II 590–668 (mean 628) neurons were included in the sample and 3.9% (3.7-4.2%) of these were GFP^+^. This result is consistent with our finding that ~29% of sst_2A_^+^ neurons in laminae I-II contain GFP, and that sst_2A_^+^ cells account for ~14% of all neurons in laminae I-II in C57Bl/6 mice [4]. It is somewhat lower than the estimate of Hantman et al. [35] that 8% of lamina II neurons were GFP^+^, and this discrepancy may have arisen because these authors did not use a stereological method. Since we have previously estimated that 24.2% of lamina II neurons are GABAergic in C57Bl/6 mice [4], the GFP cells are likely to account for ~16% of the inhibitory interneurons in this lamina.

### Responses of PrP-GFP neurons to somatostatin

Whole-cell patch-clamp recordings were performed on 17 PrP-GFP cells in lamina II. Brief subthreshold voltage steps (100 ms, -70 to -50 mV, 2.5 mV increments) were applied from a holding potential of -60 mV to establish the current–voltage (I-V) relationship of the recorded cells. The resting membrane potential and input resistance of each cell were determined from the I-V relationship. The mean resting membrane potential was -54.3 ± 2.5 mV and the input resistance was 796.6 ± 106.4 MΩ (n = 17). In current-clamp mode, each cell was injected with suprathreshold square-wave current pulses (1 s duration) to characterise its firing pattern. All of the cells were able to produce action potentials repetitively. Most (16/17) showed tonic firing, as reported previously [35], while the remaining cell was defined as an initial burst firing cell [10,54].

**Figure 6:**
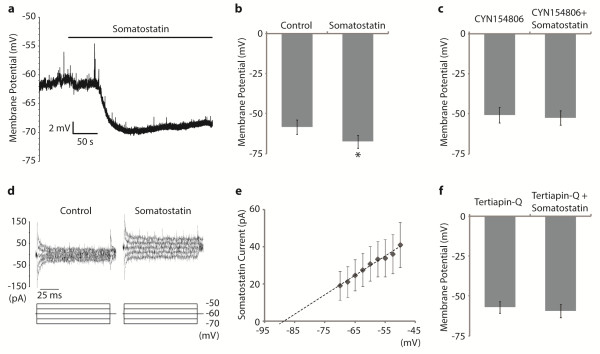
**The effect of somatostatin in PrP-GFP neurons. a** An example trace showing the membrane hyperpolarisation in response to somatostatin application. **b** The membrane potential before (control) and during somatostatin application. The mean hyperpolarisation was 8.9 ± 2.8 mV, and this was statistically significant (p < 0.05, n = 7). **c** The membrane potential did not change when somatostatin was applied in the presence of the sst_2_ receptor antagonist, CYN 154806 (n = 5). **d** Currents measured in response to brief voltage steps (100 ms, -70 to -50 mV) in control and somatostatin conditions. **e** The current–voltage (I-V) relationship for the somatostatin-evoked current (n = 7). The reversal potential of this current is approximately -90 mV. **f** The membrane potential did not change when somatostatin was applied in the presence of the GIRK channel blocker, tertiapin-Q (n = 5), suggesting that the somatostatin-mediated membrane hyperpolarisation in the PrP-GFP cells involves activation of GIRK channels.

Since all of the PrP-GFP cells were found to show sst_2A_-immunoreactivity, their response to bath-applied somatostatin was investigated. Application of somatostatin (2 μM) caused strong hyperpolarisation in all 7 PrP-GFP neurons tested (mean 8.9 ± 2.8 mV) (Figure 6a,b), which was statistically significant (p < 0.05) (Figure 6b). To assess the receptor subtype that mediated this somatostatin-evoked hyperpolarisation, a specific sst_2_ receptor antagonist, CYN 154806 (1 μM), was applied prior to and during application of somatostatin (2 μM) on a further 5 cells. In the presence of CYN 154806, the application of somatostatin did not change resting membrane potential in any of these cells (Figure 6c), indicating that sst_2_ receptors underlie the membrane hyperpolarisation.

To investigate the ionic current responsible for the somatostatin-evoked hyperpolarisation, the I-V relationship for somatostatin-evoked current was generated by subtracting the I-V plot obtained in the control recording solution from that obtained in the presence of somatostatin (n = 7, Figure 6d,e). Fitting a linear regression model, the reversal potential of this somatostatin current was approximately -90 mV, which is close to the K^+^ equilibrium potential (-97 mV) calculated by the Nernst equation (Figure 6e). Since it has been reported that the somatostatin-evoked hyperpolarising current is mediated by inwardly rectifying K^+^ channels [42,55,56], we next investigated whether GIRK channels were activated during somatostatin application. Following bath-application of the GIRK channel antagonist tertiapin-Q (0.5 μM), somatostatin (2 μM) did not alter resting membrane potentials in any of the 5 PrP-GFP cells tested (Figure 6f). Taken together, these results suggest that somatostatin acts through sst_2_ receptors, which are coupled to the activation of GIRK channels, to produce a significant membrane hyperpolarisation of PrP-GFP neurons.

## Discussion

The main findings of this study are that: (1) sst_2A_ is restricted to inhibitory interneurons in mouse superficial dorsal horn and that its expression among neurochemical subpopulations is similar to that reported in rat [13]; (2) unlike the situation in the rat [31], there is some coexistence of nNOS and galanin in superficial dorsal horn neurons in mouse; (3) all GFP cells in lamina II of the PrP-GFP mouse possess sst_2A_ receptors, and virtually all express nNOS and/or galanin; and (4) the GFP cells are all hyperpolarised by somatostatin, an effect that is prevented by application of a specific sst_2_ receptor antagonist or a GIRK-channel blocker.

### Neurochemical populations of interneurons in the mouse

It is important to identify distinct populations among the inhibitory interneurons in the superficial dorsal horn, as these are likely to have specific functions, for example preventing different types of pain [7], suppressing itch [6], or regulating other sensory inputs [36]. We have previously identified 4 non-overlapping populations among the GABAergic neurons in this region in the rat [31], and have shown that these differ in their expression of sst_2A_, which is present on approximately half of the inhibitory cells in laminae I-II [32]. Galanin- and nNOS-containing cells were found to constitute ~60% of the sst_2A_-expressing neurons in the rat, while most cells with NPY (84%) and all of those with parvalbumin did not possess sst_2A_ receptors [13]. The present results demonstrate that a similar arrangement applies in the mouse, since cells with galanin and/or nNOS again constitute ~60% of those with sst_2A_. However, there are two differences between the species: firstly, the proportion of NPY cells that express sst_2A_ is somewhat higher (24% in mouse, compared with 16% in rat [13]), and secondly there is a significant population of cells that contain both galanin and nNOS in the mouse. These cells correspond to 30% of those with galanin and 56% of those with nNOS, and they generally contain relatively low levels of both substances.

There is already evidence that these neurochemically defined populations differ in their postsynaptic targets. We have reported that in the rat, GABAergic axons containing NPY preferentially innervate lamina III projection neurons that express the neurokinin 1 receptor (NK1r) [57,58], while some of those that contain nNOS are presynaptic to giant lamina I spinoparabrachial neurons [59]. It has also been shown that the parvalbumin-containing inhibitory interneurons, most of which are islet cells [60], give rise to axoaxonic synapses on low-threshold myelinated primary afferents, and these cells are therefore presumably involved in the regulation of tactile inputs [36]. In the case of the NPY innervation of lamina III projection neurons, this is thought to arise from a specific subset of NPY-containing inhibitory interneurons [57], and a similar arrangement may also apply to the nNOS inputs to the giant lamina I cells. If this is the case, it would suggest that the populations defined by these neurochemical markers are not homogeneous, but are likely to contain further subpopulations that differ in connections, and therefore function.

### PrP-GFP cells and the nNOS and galanin populations of inhibitory interneurons

In a series of elegant studies, Perl and colleagues have described the physiological properties of the PrP-GFP cells in detail [34,35,40]. These cells receive monosynaptic primary afferent input from a class of relatively fast-conducting C fibres, but not from Aδ afferents. They also show specific patterns of connection to other neurons in lamina II, since they can be reciprocally connected to islet cells (which are also GABAergic), and can be presynaptic (but not postsynaptic) to excitatory vertical cells [40].

These studies had already provided evidence that at least the majority of the PrP-GFP cells were inhibitory, as GABA immunoreactivity was seen in >80% of them [35], and they invariably generated IPSCs in the postsynaptic cell in paired recordings [40]. Here we show that all of these cells are inhibitory, since they all express sst_2A_, which is restricted to GABA-immunoreactive neurons in lamina II. As stated by Hantman et al. [35], it is likely that their failure to detect GABA in all of the PrP-GFP cells was due to limited penetration of GABA-immunostaining. This was not an issue in the present study, as our assessment of GABA in sst_2A_^+^ cells was restricted to those located at the section surface.

Our immunocytochemical findings with the PrP-GFP cells further support the neurochemical classification scheme outlined above, since the GFP cells were entirely contained among those inhibitory interneurons that express sst_2A_[4], and within this group, they corresponded to part of the set that contained nNOS and/or galanin. The expression of GFP among the nNOS- and galanin-containing inhibitory interneuron populations was not random, as GFP cells accounted for less than a quarter of the galanin^+^/nNOS^-^ cells, over half of the galanin^-^/nNOS^+^ cells, and the great majority (>80%) of those that contained both nNOS and galanin. In addition, the strength of GFP labelling tended to be higher in the nNOS-containing cells.

Although both nNOS- and galanin-containing inhibitory interneurons express sst_2A_, several features indicate that in the rat there are significant differences between them. Glycine is often present at high levels in the nNOS cells, but is not enriched in the galanin-containing cells [51,61]. This suggests that nNOS (but not galanin) cells may use glycine as a co-transmitter, although it is not known whether somatic glycine enrichment is a reliable marker for glycinergic neurons [39]. In addition, although studies with the transcription factor Fos indicate that both types can respond to noxious stimuli, the nNOS cells appear far less likely to phosphorylate extracellular signal-regulated kinases than the galanin cells, and are less responsive to subcutaneous injection of capsaicin [13]. However, the present results indicate that there are common features between nNOS and galanin populations in the mouse, since some cells can contain both substances, and the PrP-GFP cells include those with nNOS and/or galanin. The Allen Brain atlas shows that although cells with mRNA for galanin are concentrated in lamina I and the outer part of lamina II in adult mice (matching the pattern seen with immunocytochemistry), they are more widely distributed at P4, with numerous cells located in the inner half of lamina II and lamina III. In contrast, expression of nNOS by neurons in laminae I-III appears relatively late in development, with the adult pattern only being established by the end of the third postnatal week in the rat [62]. This raises the possibility that some inhibitory interneurons may initially contain galanin, and then switch to nNOS expression during postnatal development. If so, then this mechanism may be more effective in the rat, whereas in the mouse some cells continue to express both nNOS and galanin, but with relatively low levels of each. We have recently found that in mice lacking the transcription factor Bhlhb5 [6], there is a substantial loss of cells that contain nNOS and/or galanin, but not of other inhibitory interneurons, (AJT, EP, D Cameron and SE Ross, unpublished data), which supports the suggestion that the nNOS and galanin cells are developmentally linked.

As stated above, studies with activity-dependent markers have suggested that there are differences in the responsiveness of nNOS- and galanin-containing cells in the rat superficial dorsal horn to different forms of noxious stimulation [13]. Both populations seem to be activated by noxious heat and subcutaneous formalin, while the galanin (but not the nNOS) cells also respond to capsaicin. It will therefore be of interest to determine in future studies whether there are similar differences between these neurochemical populations in the mouse, and if so, how the cells that co-express nNOS and galanin respond to these stimuli. It will also be important to see whether there are differences in the types of primary afferent that innervate these populations.

### Role of somatostatin

Our pharmacological results are consistent with the anatomical finding of sst_2A_ expression by PrP-GFP cells, since all of the GFP neurons tested were hyperpolarised by bath-applied somatostatin, and this effect was prevented by a specific sst_2_ antagonist. Kim et al. [42] had previously reported that the somatostatin-evoked hyperpolarization of lamina II neurons involved GIRK channels, and our finding that the action of somatostatin on the GFP cells was blocked by tertiapin-Q supports this conclusion. Further evidence for the specificity of somatostatin comes from the observation that it does not cause outward currents when applied to excitatory interneurons in lamina II of the rat [14]. This consistency between anatomical and pharmacological results is in contrast to findings with the μ-opioid receptor MOR-1, since MOR-1-immunoreactivity is apparently restricted to a small proportion (~10%) of lamina II neurons [63], whereas hyperpolarising effects of μ-opioid agonists are seen on a far higher proportion (often more than 50%) of neurons recorded in both lamina I and II [64-67].

Among the 5 somatostatin receptors, only sst_2_ appears to be expressed by neurons in the mouse superficial dorsal horn (Allen Brain atlas), although both sst_1_ and sst_2_ receptors are present on small and medium-sized (presumed nociceptive) primary afferents [68,69]. Several previous studies have reported that sst_2A_ receptors were concentrated in the superficial dorsal horn [4,32,68-70], and we have demonstrated that in both rat and mouse these are virtually restricted to GABAergic interneurons [4,14,32] (present study). Since somatostatin exerts an inhibitory action, activation of sst_2A_ receptors on these interneurons should lead to disinhibition, and is therefore likely to increase sensory transmission, for example resulting in increased pain or itch [14]. In contrast, activation of somatostatin receptors on primary afferents would suppress sensory transmission. It is therefore not surprising that both pro-nociceptive [71-73] and anti-nociceptive [74-76] effects of intrathecal somatostatin have been reported. In fact, the main behavioural sign that was seen following intrathecal administration of a low dose of somatostatin was caudally directed biting and scratching, and although this was interpreted as increased pain [71-73], it could also have been caused by increased itching.

The somatostatin that acts on the sst_2A_ receptors expressed by the GFP cells could originate from primary afferents [77] and/or excitatory interneurons in lamina II [78]. Nakatsuka et al. [56] were able to evoke a GIRK-mediated hyperpolarisation in some lamina II neurons, which was thought to be caused by somatostatin, as it was partially occluded by somatostatin-evoked currents, and was blocked by the non-specific antagonist cyclo-somatostatin. This hyperpolarisation was consistently obtained by focal electrical stimulation in the dorsal horn, but not by dorsal root stimulation, which suggests that the excitatory interneurons may be a more important source of somatostatin acting on these receptors, contributing to the intrinsic modulation of the neuronal network in the superficial dorsal horn. Hantman and Perl [34] have shown that all the PrP-GFP cells tested were hyperpolarised by both norepinephrine and serotonin, whereas varied responses were observed for other lamina II neurons. Taken together with the present results, this indicates that the PrP-GFP cells show consistent responses to a variety of neuromodulators.

## Conclusions

These results demonstrate that the neurochemical organisation of inhibitory interneurons in the superficial dorsal horn of the mouse is similar to that previously reported for the rat. The finding that all GFP^+^ neurons in the PrP-GFP mouse possess sst_2A_ receptors, and that they express nNOS and/or galanin supports the suggestion that neurochemistry provides a useful way of defining functional populations among the interneurons in the superficial laminae. The present findings, taken together with previous reports that the PrP-GFP cells form a relatively homogeneous population in terms of their physiological properties, suggest that these cells may have discrete functional roles in processing somatosensory information.

## Abbreviations

GFP: Green fluorescent protein; GIRK: G-protein-coupled inwardly rectifying K^+^; NA: Numerical aperture; nNOS: Neuronal nitric oxide synthase; NPY: Neuropeptide Y.

## Competing interests

The authors declare that they have no competing interests.

## Authors’ contributions

NI was involved in design of the electrophysiological part of the study, performed the patch-clamp experiments and analysed the resulting data; FG participated in the anatomical studies and analysed the data; EP contributed to the anatomical studies; JSR helped design the electrophysiological experiments and contributed to data interpretation; AJT conceived the study, participated in the design, experiments and analysis, and drafted the manuscript. All authors read and approved the final manuscript.
